# A Low-Cost Three-Dimensional Printed Retractor for Transforaminal Lumbar Interbody Fusion

**DOI:** 10.7759/cureus.24185

**Published:** 2022-04-16

**Authors:** Manuel de Jesus Encarnacion Ramirez, Renat Nurmukhametov, Edwin Bernard, Ismael Peralta, Ibrahim E Efe

**Affiliations:** 1 Department of Neurosurgery, Peoples' Friendship University of Russia, Moscow, RUS; 2 Division of Spine Surgery, Central Clinical Hospital of the Russian Academy of Sciences, Moscow, RUS; 3 Department of Neurosurgery, Hospital Regional Alejandro Cabral, San Juan de la Maguana, DOM; 4 Department of Neurosurgery, Charité - Universitätsmedizin Berlin, Berlin, DEU

**Keywords:** lumbar spine, tlif, low cost, retractor, 3d modeling, 3d printing

## Abstract

The authors developed a low-cost surgical retractor to improve surgeon’s comfort and facilitate pedicle screw insertion in transforaminal lumbar interbody fusion surgery. The retractor was designed using three-dimensional (3D) modeling software and produced with the help of a 3D printer. It was attached to a mechanic retractor arm. The retractor was anchored to the transverse process through a concave notch at its tip, visualizing the junction between the transverse process and the superior articular process. The gutter-shaped body of the retractor helped stay within the ideal trajectory during screw insertion. The retractor was tested in 20 patients undergoing transforaminal lumbar interbody fusion with satisfactory results. Future models will be generated suitable for surgery of the cervical and thoracic spine.

## Introduction

In recent years, three-dimensional (3D) printers have become increasingly accessible and proven useful in a wide range of neurosurgical applications [1,2]. The rapid prototyping of surgical instruments and implants allows for personalized and low-cost solutions [3]. Surgeons in low- and middle-income countries need to cope with the lack of appropriate equipment or timely access thereto. Thus, 3D printing holds the promise of bridging the gap between the surgeon and the industry in low-resource environments.

Crooks et al. identified the need for better thumb stabilization during surgery for acute hand trauma. Using 3D printing, they successfully developed a hand retractor allowing optimal access to the surgical site. Their retractor can be printed in different sizes accommodating a wide range of patients [4]. Rankin et al. produced army/navy retractors from polylactic acid filament. Their model met the demands of the conventional stainless steel equivalent at only one-tenth its cost and only 90 minutes of printing duration [3].

We previously used manual retractors in transforaminal lumbar interbody fusion surgery (TLIF). Not only did these retractors only serve the purpose of retracting soft tissue but also led to surgeons’ fatigue. We, hence, explored the potential of 3D modeling and printing to design a versatile alternative. In this report, we introduce a novel low-cost 3D-printed retractor for TLIF surgery that can: (a) increase surgeon’s comfort; (b) help visualize landmarks and identify the ideal screw entry point; (c) guide the ideal screw trajectory; (d) protect paraspinal muscles.

## Technical report

**Figure 1:**
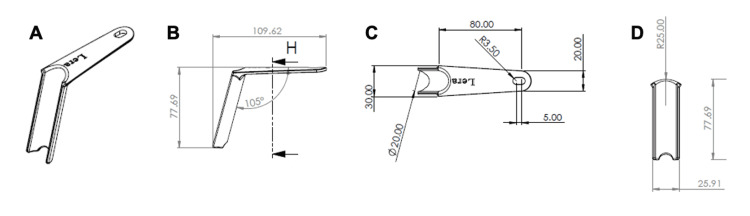
Technical drawings showing the LERA retractor (A) in the side view (B), top view (C), and frontal view (D). Lengths are shown in millimeters. LERA: Lumbar Expander/Retractor Assistant

The retractor was designed with the help of the AutoCAD and MeshMixer software (Autodesk Inc., San Rafael, California, United States). The retractor is gutter-shaped to serve as a guide rail during screw insertion. It features a concave notch at its tip that can be anchored to the transverse process (Figure 1).

The digital model was converted into a 3D-printable object using the CHITUBOX program (Shenzhen, Guangdong, China). The retractor was 3D-printed using the Kelant S400 printer (Shenzhen Kelant Technology Co. Ltd., Shenzhen, Guangdong, China) and Formlabs dental model resin (Formlabs Inc., Somerville, Massachusetts, United States). The resin met all biosafety level requirements for use in the human body. It was sterilized through low-temperature sterilization using ethylene oxide. The finished product was named “Lumbar Expander/Retractor Assistant” (LERA). Printing one LERA retractor took a maximum of three hours. The cost of one liter of resin was roughly US$ 185. A minimum of 25 LERA retractors can be produced from one liter of resin.

**Figure 2:**
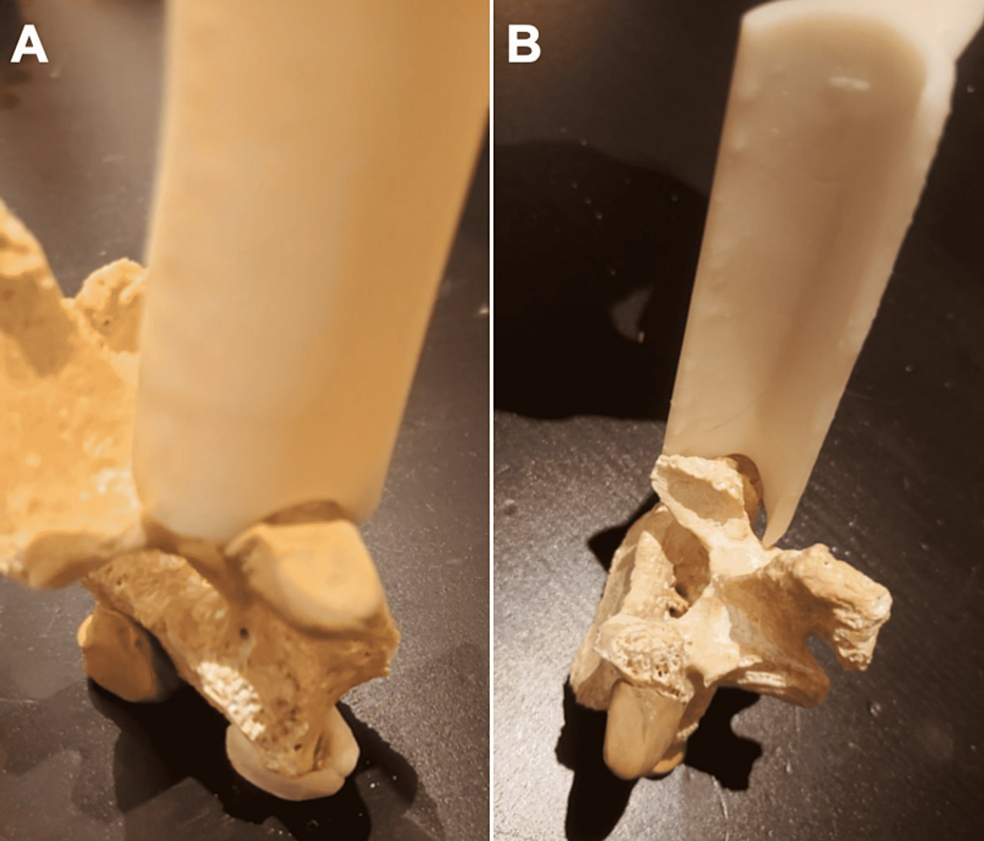
The LERA has a concave notch through which it can be anchored to the transverse process (A). The end of the gutter-shaped retractor points at the junction between the transverse process and the superior articular process, centering the screw entry point (B). LERA: Lumbar Expander/Retractor Assistant

Twenty patients underwent LERA-assisted TLIF. Fourteen patients had two-level surgery and six patients had three-level surgery. Patients were operated on in the prone position. A posterior midline incision was made. Superficial and deep fascias were opened and the paravertebral muscles were dissected away from the spine. The transverse process was identified through palpation. The vertebral segment was checked on x-ray before inserting the LERA retractor. The concavely curved tip was anchored to the transverse process, pointing at the junction between the transverse process and the superior articular process (Figure 2).

**Figure 3:**
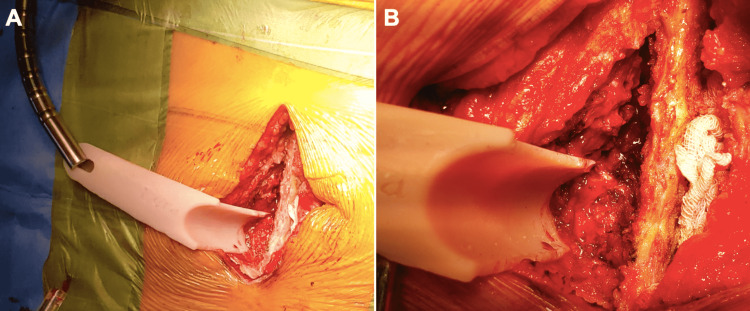
The LERA retractor was secured to a flexible retractor arm through a mounting hole (A). During the TLIF procedure, the retractor aimed at the junction between the superior articular process and the transverse process, protecting the paraspinal muscles (B). LERA: Lumbar Expander/Retractor Assistant; TLIF: transforaminal lumbar interbody fusion surgery

 The LERA was then secured to an automatic retractor arm, exerting tension onto the paraspinal muscles (Figure 3).

**Figure 4:**
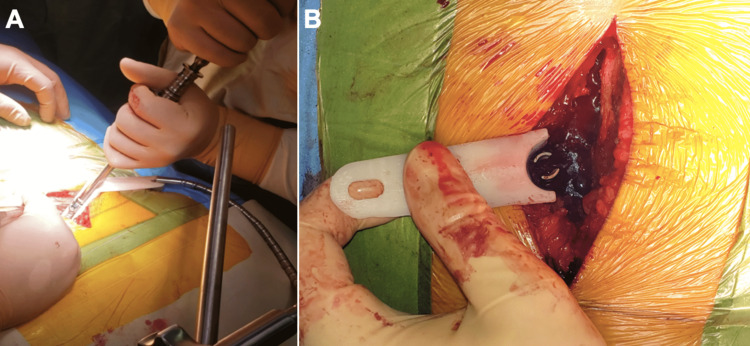
The LERA retractor can help guide the pedicle screw trajectory (A). In the absence of a flexible retractor arm, the retractor can be used manually (B). The inserted pedicle screw can be appreciated. LERA: Lumbar Expander/Retractor Assistant

Alternatively, it can be used for dynamic retraction in a hand-held manner. Laminectomy and facetectomy were performed. The cord was decompressed, and the nerve roots were visualized. The anulotomy was performed, followed by a complete discectomy and endplate preparation. Bone autograft and a polyetheretherketone (PEEK) cage were used for interbody fusion. The cortical bone was removed at the screw entry point using a Kerrison rongeur. Bilateral pedicle screws and rods were inserted using standard anatomic landmarks (Figure 4).

Fluoroscopy was used to control the screw position after insertion. The retractor is radiolucent, allowing C-arm use with no need to remove the retractor. Patients were advised to start walking on the day after surgery and were usually discharged on postoperative day two or three. Pain medication and muscle relaxants were prescribed. The patients were routinely evaluated by the surgeons at intervals of one, three, six, and 12 months after surgery. All patients who underwent LERA-assisted TLIF showed satisfactory symptom relief comparable to those who underwent conventional TLIF in our center. 

## Discussion

To the best of our knowledge, no similar resin-based 3D-printed surgical retractor has been published to this date. Resin materials have previously proven high-wear resistance in 3D printing-based dental prosthesis manufacturing [5]. Our retractor described in this report withstood all 20 surgeries with no sign of wear. However, biomechanical stress tests were not conducted to evaluate whether it could tolerate forces beyond those necessary in spine surgery. Previous literature advised against the use of steam sterilization methods for 3D-printed materials due to their high heat intolerance. Low-temperature sterilization techniques are a common alternative to prolong the durability of sensitive materials [6]. Testing was done with five-minute steam sterilization at a maximum temperature of 134°C and no structural damage or relevant reduction in mechanical resistance of the LERA was observed. Nevertheless, low-temperature sterilization with 100% ethylene oxide was chosen as the standard protocol. Ethylene oxide is an FDA-approved gaseous sterilizing agent. Yet, high levels of ethylene oxide residue on the instrument may be hazardous to the patient. Glutaraldehyde, hydrogen peroxide gas plasma, and ozone may be safer alternatives [3,6]. 

The LERA led to a marked improvement in surgeon’s comfort compared to hand-held retractors. Further, it helped better visualize the confines of the superior articular process and to identify the ideal screw entry point. Among the younger staff, in particular, the LERA helped stay within the intended trajectory during screw insertion. 

The LERA retractor’s dimensions, however, are suited exclusively for conventional non-minimally invasive surgery of the adult lumbar spine. Even in cases of altered anatomy due to, for example, marked obesity, facet hypertrophy, or a displaced fracture, the LERA may not be suitable. The digital 3D modeling tools and printers, however, allow personalized customization of the retractor design to overcome this limitation. Although the LERA itself is a very low-cost tool, it can only unfold its full potential when attached to a mechanic retractor arm. 

Surgeons interested in adopting 3D printing technology should consider the purchasing costs of commercially available 3D printers and 3D modeling software licenses. Despite rapidly decreasing costs of 3D printing hardware, considerable cost savings are only achieved after frequent use. Further, beginners should not underestimate the learning curve that must be overcome to accurately generate 3D-printed objects [7]. 

The LERA retractor is only one of numerous examples of the wide-ranging surgical applications that arise from 3D printing. We aim to design similar retractors for other spinal indications and the cervical and thoracic segments of the spine in the near future. Through patient surveys, we will also assess the LERA retractor’s effect on postoperative pain.

## Conclusions

The LERA is a versatile low-budget retractor that can help better visualize anatomic landmarks while providing protection to the surrounding soft tissue. Attached to a mechanic retractor arm, it can significantly improve surgeon’s comfort. Especially for residents and young neurosurgeons, the LERA can help by guiding the screw trajectory. The authors aim to generate further models for the cervical and thoracic spine.
